# Multiple sampling schemes and deep learning improve active learning performance in drug-drug interaction information retrieval analysis from the literature

**DOI:** 10.1186/s13326-023-00287-7

**Published:** 2023-05-30

**Authors:** Weixin Xie, Kunjie Fan, Shijun Zhang, Lang Li

**Affiliations:** Department of Biomedical Informatics, Ohio State University, Columbus, OH 43210 USA

**Keywords:** Active learning, Deep learning, Drug-drug interaction, Information retrieval, Random negative sampling, Positive sampling, Similarity sampling, Uncertainty sampling

## Abstract

**Background:**

Drug-drug interaction (DDI) information retrieval (IR) is an important natural language process (NLP) task from the PubMed literature. For the first time, active learning (AL) is studied in DDI IR analysis. DDI IR analysis from PubMed abstracts faces the challenges of relatively small positive DDI samples among overwhelmingly large negative samples. Random negative sampling and positive sampling are purposely designed to improve the efficiency of AL analysis. The consistency of random negative sampling and positive sampling is shown in the paper.

**Results:**

PubMed abstracts are divided into two pools. Screened pool contains all abstracts that pass the DDI keywords query in PubMed, while unscreened pool includes all the other abstracts. At a prespecified recall rate of 0.95, DDI IR analysis precision is evaluated and compared. In screened pool IR analysis using supporting vector machine (SVM), similarity sampling plus uncertainty sampling improves the precision over uncertainty sampling, from 0.89 to 0.92 respectively. In the unscreened pool IR analysis, the integrated random negative sampling, positive sampling, and similarity sampling improve the precision over uncertainty sampling along, from 0.72 to 0.81 respectively. When we change the SVM to a deep learning method, all sampling schemes consistently improve DDI AL analysis in both screened pool and unscreened pool. Deep learning has significant improvement of precision over SVM, 0.96 vs. 0.92 in screened pool, and 0.90 vs. 0.81 in the unscreened pool, respectively.

**Conclusions:**

By integrating various sampling schemes and deep learning algorithms into AL, the DDI IR analysis from literature is significantly improved. The random negative sampling and positive sampling are highly effective methods in improving AL analysis where the positive and negative samples are extremely imbalanced.

**Supplementary Information:**

The online version contains supplementary material available at 10.1186/s13326-023-00287-7.

## Background

Drug-drug interaction (DDI) is one of the major risk factors that cause adverse drug events (ADEs). Nearly 22% and 9% of ED visits and hospitalizations, respectively, are caused by DDIs [1–4]. DDIs are most prevalent among older adults because of the disproportionately high prevalence of polypharmacy [5–7]. DDI is a major research topic in pharmacokinetics (PK) and pharmaco-epidemiology (PE) studies. The DDI pharmacology mechanisms usually are investigated in PK studies, in which the change of one drug’s metabolism and transportation are compared in the presence and absence of another drug [8]. PK DDI studies sometimes are performed in vitro, i.e. using either recombinant enzymes, or human liver microsome, or hepatocyte. Clinical PK DDI study is another important approach in assessing whether one drug exposure is altered by another co-committed drugs [8, 9]. Using large scale claim or EHR databases, pharmaco-epidemiological studies, on the other hand, focus on whether DDIs change ADE risks in targeted patient populations [10]. DDI induced ADEs, sometimes, are also published in patient case reports [11, 12]. If drug combinations are tested in controlled clinical trials, their efficacy and ADEs are always compared to those of single drugs [13]. An enormous amount of DDI information has been published in the biomedical literature. It has been a great interest in mining and curating these DDI information for assisting physicians and patients in preventing DDIs and their associated ADEs [14]. In this paper, we will focus on mining published DDI studies related to ADEs. They are either pharmacoepidemiology studies, or case reports, or controlled clinical trials [15].

There are two major DDI text mining tasks from PubMed: information retrieval (IR) [16] and information extraction (IE). The goal of DDI IR is to identify DDI relevant publications or abstracts, while DDI IE is to extract DDI pairs from the DDI relevant publications or abstracts [17]. DDI IR is always the first step in identifying DDI relevant publications and abstracts. Then, DDI IE task relies on annotated DDI relationships in positively labeled DDI paper or abstracts generated from the DDI IR step. DDI IR and IE analyses were reviewed in our early paper in 2014 [18]. In this paper, our literature review will focus on DDI IE and IR methods after 2014.

Deep learning (DL) techniques are clearly the major trend in recent DDI IE analysis. Zhao et al. [19] proposed a syntax convolutional neural network that combined a traditional convolutional neural network and external features (contexts, shortest path, part-of-speech) to extract DDIs. It obtained a F1-scores of 0.69 for DDI extraction. By integrating a recurrent neural network with multichannel word embedding, Zheng et al. [20] combined an attention mechanism and a recurrent neural network with long short-term memory (LSTM) units and obtained a system that performed well for DDI extraction (F1 = 0.77). Zhang et al. [21] integrated the shortest dependency paths and sentence sequence by a hierarchical recurrent neural networks-based method, which produced an F1-score of 0.73 for DDI extraction. Wang et al. [22] introduced the dependency-based technique to a bi-directional LSTM network, built a linear depth-first search and a breadth-first search, and it achieved an F1-score of 0.72 for DDI extraction. In a recent paper, Zhang et al. [23] shortest dependency path was integrated with both convolutional neuron network model and recurrent neuron network model in DDI IE analysis. It reported an F1-score of 0.75. Recently, utilizing drug information from drug database increased the DDI IE performance from the literature [24, 25].

However, the research on DDI IR analysis has not been as advanced as DDI IE methodology development. Our DDI IR analysis in 2015 presented the most comprehensive comparisons among many machine learning (ML) methods. It demonstrated that linear discriminant analysis, logistic regression, and supporting vector machine all had similar performance, F1-score = 0.93, in identifying DDI related abstracts in PubMed [26]. However, if the recall rate was set as 0.95, DDI IR precision became as low as 0.67.

The under-developed DDI IR methodology is largely due to the lack of negatively labeled DDI PubMed in the existing DDI corpora [27] which contain only positively label DDI abstracts, including our recently published corpus [28]. While building up more negatively and positively labeled DDI abstracts shall certainly help in further developing DDI IR methodology, it is more interesting to explore the interactive process between DDI annotations and DDI IR optimization. This falls into one territory of artificial intelligent field, active learning (AL) [29]. AL attempts to maximize the performance of the ML algorithms while annotating as few samples as possible [30]. The application of AL to biomedical text mining is rather limited. As one example, its use to mine text in electronic medical record data to identify disease phenotype reduced the number of annotated samples required to achieve an AUC of 0.95 by 68% in predicting patients with rheumatoid arthritis.

Introduced by Lewis and Gale in 1994 [31], AL optimizes ML algorithms sequentially based on user feedback. AL uses uncertainty sampling to guide ML training on new samples for which ML has demonstrated the lowest predictive performance. The primary AL research has focused on uncertainty sampling schemes, such as least confidence, margin sampling, entropy, query by committee, expected model change, expected error reduction and variance reduction [32]. Although AL can potentially improve the DDI IR analysis, there are several challenges that motivate the development of new AL methodology in this paper. Firstly, positively labeled DDI abstracts in the current DDI corpora were selected from a query of keywords, such as “drug interaction,” limits the identification of all relevant general abstracts in PubMed. If this is ignored in AL, it will lead to a biased DDI IR analysis. Secondly, more than 99% of PubMed abstracts are unrelated to DDIs, and the labeling of positive samples is more labor intensive, and therefore more expensive than labeling negative samples. Thus, to be more cost effective, AL should take greater advantage of the large-scale availability of negative data, but current uncertainty sampling schemes do not deal with them. DL approaches have been developed and implemented for the DDI IE analysis, but not yet for DDI IE. DL could significantly improve the performance of AL with respect to DDI IR.

## Methods

### DDI corpus and annotation guideline

There are two sample pools in this study. The first one is called screened sample pool, which are the abstracts in PubMed through keyword queries: [“drug interaction” AND (Type of Study)] and [“drug combination” AND (Type of Study)]. The “Type of Study” is defined in Table 1: clinical trial, pharmaco-epidemiology study, and case report. Based on the criteria for DDI abstract selection in Table 1, sample abstracts are reviewed and annotated. A corpus is built, which has 933 positive DDI abstracts and 799 negative abstracts. They are the initial labeled samples in the screened sample pool. Table 1 presents inclusion and exclusion criteria for the screened sample pool abstract selection. 5,000 abstracts are randomly selected from screened samples as the screened sample pool in this study.

The other sample pool is called unscreened sample pool. It is made up of 10,000 abstracts that are randomly selected from PubMed and are not overlapped with screened sample pool. This unscreened sample pool, on the other hand, contains data are largely not DDI relevant. Data distribution for screened sample pool and unscreened sample pool is shown in Table 2.

Two annotators with complementary skills in biology and informatics develops this corpus. Mrs. Shijun Zhang, has a master’s degree in biology, and has worked in Dr. Li’s lab for 7 years with the primary research responsibility of corpus development for drug-interaction text mining [27]; Mrs. Weixin Xie, a PhD student in medical informatics, has conducted pharmacology and drug-interaction text-mining research under Dr. Li’s supervision. Training and education in labeling have an initial calibration step, in which two individuals label each abstract according to the inclusion and exclusion criteria outlined in Table 1 [28], the agreement between their labels is then evaluated for the first 30 positive abstracts (30 in each of the three DDI categories), and they receive further training based on that analysis.

**Table 1:** Inclusion and exclusion criteria for clinical DDI abstract selection

**Inclusion (positive)**	Clinical trial DDI study: Phase I/II/III clinical trials in which drug combination and/or single drug ADE are evaluated and reported.
	Pharmaco-epidemiological DDI study: Pharmaco-epidemiology studies in which ADEs from drug combinations are reported and compared to single drug ADEs.
	DDI and ADE case reports: DDI-induced ADE cases in which the time sequential drug and ADE are reported in clinical care settings.
**Exclusion (negative)**	Clinical PK DDI study: both single drug and drug combination exposures (i.e. pharmacokinetics) are evaluated either in patients or healthy volunteers.
	Clinical PK PG study: the single drug exposure (i.e. pharmacokinetics) is evaluated among patients that have different genotypes in CYP450 and UGT enzymes and drug transporters.
	in vitro PK study: substrate depletion and metabolite formation study is for the fm data collection; and inhibition study is for the Ki data collection.
	Drug interaction detection algorithms or software
	Compliance of avoiding DDI
	Concordance of DDI reporting among different drug interaction knowledge base.
	Comparison of the performance of DDI clinical decision systems
	Drug-alcohol/food interactions
	Drug/test interactions
	Case report studies
	Review papers
	Cell culture and animal studies
	Other studies that are not related to drug interactions.

**Table 2:** Statistics of DDI corpus

**Data Source**	**Sample pool**	**Data set**	**Sample size**	**Initial training set**	**Initial validation set**
PubMed	Screened sample pool	Labeled Positive	150	100 +*	50 +*
		Labeled Negative	799	100 -*	50 -*
		Unlabeled screened samples	3,169		50 R*
	Unscreened sample pool	Unlabeled unscreened samples	9,999	100 +*	50 +*
				100 R*	50 -*
					50 R*

+* (labeled positive samples), -* (labeled negative samples), R* (random negative samples)

### Sampling strategies in active learning

- *Uncertainty sampling* in AL refers to selecting the least confidence new samples, e.g. abstracts with predicted probability around 0.5 in a binary classification (i.e. DDI relevant or not), for the next round labeling and training in machine learning analysis.
- *Positive sampling* refers to selecting the most certain positive new samples, e.g. predicted probability close to 1 in a binary classification, for next round labeling and training in machine learning analysis. Positive sampling is absolutely necessary in the unscreened sample pool because most of samples are negative. In the screened sample pool, as most of samples are positive, positive sampling scheme is not needed.
- *Random negative sampling* Because more than 99% of unscreened pool abstracts are not DDl related, a random subset of unscreened pool is chosen as negative samples. These random negative samples may contain very small fraction of positive samples [33].
- *Similarity sampling* *aims to quick screen out samples that more like samples in corpus*, the cosine similarity (cosSIM) based on TF-IDF (Term Frequency-Inverse Document Frequency) [34] of each unlabeled sample and all the samples in corpus is used to evaluated. The TF(t) and IDF(t) of term t (word t) are formulated as

$$\begin{array}{cc}TF\;(t)\;=\;\frac{term\;t\;\#\;\;in\;an\;abstract}{total\;\;term\;\#\;in\;an\;abstract};&IDF(t)=In\frac{Total\;\#\;of\;abstract}{\#\;of\;abstract\;with\;term\;t}\;;\end{array}$$

TF(t) measures how frequently a term *t* occurs in an abstract, and *IDF(t)* measures how important the term t is. In fact, certain terms that occur too frequently have little power in determining the relevance, therefore, we need to weigh up the effects of the less frequently occurring terms. And then, we got the TFIDF for term t by computing the following:

$$TFIDF\left(t\right)=TF\left(t\right)\times IDF\left(t\right);$$

Above multiplying TF(t) and IDF(t) results in the TFIDF score of a term t in an abstract. The higher the score, the more relevant that term is in that particular abstract. For each abstract, we derived 30 key terms with high TFIDF, and their frequency vector of each abstract was generated to calculate the cosine similarity (cosSIM). For example, abstracts A and B are two n-dimensional vectors, $A=({A}_{1},\dots ,{A}_{n})$ and $B=({B}_{1},\dots ,{B}_{n})$, using the formula below we can find out the cosine similarity between *A* and *B*: 

$$cosSIM\left(A,B\right)=\frac{\vert A\cdot B\vert}{\left|A\right|\times\left|B\right|}.$$

Here, the cosine similarity of abstract A and B ranges from 0 to 1. In this study, sample in pools has its similarity values with samples which are DDI related abstracts, the higher the similarity value, the more DDI related abstract likely. Similarity sampling is applied in conjunction with other sampling strategies in two sample pools, they will benefit to training models.

Existing AL analyses only uses uncertainty sampling. In this paper, we will study whether random negative sampling, positive sampling and similarity sampling will increase the performance of AL analysis.

### Active learning with random negative sampling converges to the same optimal classifier as active learning

In our AL analysis, the absence of manual labeling reduces the expense involved with negative random negative sampling, but a small fraction of mislabeled negative samples requires correction to avoid classifier bias. Through the iterative AL process, we expect the asymptotic reduction of this bias to zero as the sample size grows. However, this random negative sampling scheme is beyond the scope of the current AL framework [35, 36], which allows no mislabeled samples. Here is a heuristic proof to clarify the convergence of AL with negative random negative sampling to the same AL optimal classifier.

Let us use a similar notion to that of Balcan and Long [37]. We assume that the data points $(x, y)$ are drawn from an unknown underlying distribution ${D}_{XY}$ over $X\times Y$. $X$ is called the feature space (e.g. word frequencies in abstracts), and $Y$ is the abstract label. Here, $Y=\left\{\pm 1\right\}$ and $X={\mathbb{R}}^{d}$, and $d$ is the dimension. Without loss of generality, we further assume that the feature space *X* is centralized in 0 after linear transformation. Let $\mathbb{C}$ be a class of linear classifiers through the origin, that is $\mathbb{C}=\left\{sign\left(w\bullet x\right):w\in\mathbb{R}^d,\left\|\mathrm w\right\|=1\right\}$. In an AL, the goal is to identify a classifier $w\in \mathbb{C}$ of small misclassification error, where $err\left(w\right)={P}_{\left(x,y\right)\tilde{D}_{XY}}\left[sign\right(w\bullet x)\ne y]$. Balcan and Long showed that with arbitrary small error $\in$ and probability $\delta$, an AL needs at most $O\left(\left(d+\text{log}(1/\delta)+loglog\left(1/\in\right)\right)\text{log}(1/\in)\right)$labeled samples to identify a classifier with misclassification error less than $\in$ and probability higher than $1-\delta$. This AL theory requires no misclassification error among sample labels.

In the unscreened sample pool, let us assume the mislabeling negative sample size,${n}_{\mp }$, is much smaller than negative samples in random negative sampling ${N}_{-}$, i.e. ${n}_{\mp }\ll {N}_{-}$. The true positive samples in the training set,${N}_{+}$ is also smaller than ${N}_{-}.$ Therefore, the error rate before the AL classifier is approximated in Eq. (1). Using the AL classifier, there will be ${n}_{\mp }\times {N}_{+}/({N}_{-}+{N}_{+})$ mislabeled negative samples predicted to be positive, and their labels will be calibrated through the manual label in the AL. After AL calibration, the error rate of will be reduced to Eq. (2).

1 $$Error\;rate\;before\;AL:\;\epsilon\frac{n_\mp}{N_-+N_+};$$

2 $$Error\;rate\;after\;AL\;\epsilon\;+\;\frac{n_\mp}{N_-+N_+}\;\times\;\frac{N_++n_\mp\times{\displaystyle\frac{N_+}{N_-+N_+}}}{N_-+N_+}.$$

Practically, considering, ${n}_{\mp }={N}_{-}/1000$ and ${N}_{+}={N}_{-}/4$. The error is $\in+0.001$ before AL, and $\in+0.001/5$ after one step AL calibration, and $\in+0.001/5^m$ after m steps. Therefore, the misclassification error due to the mislabeled data will go to zero extremely fast. This heuristic proof has not yet considered the complications such as nonlinear classified, general log-concave distributions, and inseparable positive and negative data in ${\mathbb{R}}^{d}$. Existing AL theories [37, 38] have shown and supported that error $\in$ holds with required $\left(\left(d+\text{log}(1/\delta)+loglog\left(1/\in\right)\right)\text{log}(1/\in)\right)$labeled samples under these conditions. We, however, will use the similar argument to show that the mislabeled error ${n}_{\mp }/({N}_{-}+{N}_{+})$ will become small after a number of AL steps.

### Positive sampling improves AL optimization when sample population is overwhelming negative

Following the same annotation, $\in$ is the prespecified misclassification error. Collecting positively labeled samples is not an easy task using uncertainty sampling alone when sample population is overwhelming negative. Here, population positive sample size ${N}_{+}$ is significantly smaller than population negative sample size, ${N}_{-}$. The misclassification error rate of negative samples is $\in\times\frac{N_-}{N_-+N_+}$, while the misclassification error of positive samples is $\in\times\frac{N_+}{N_-+N_+}$. Given a positive sample in the sample pool available for selection, uncertainty sampling focuses on misclassified samples, and it has a $\in\times\frac{N_+}{N_-+N_+}$ chance in selecting this positive sample. On the hand, in positive sampling, top $\alpha$, a percentage, positively predicted samples will be selected. Hence, positive samples have $\left(1-\in\times\frac{N_+}{N_-+N_+}\right)\times\alpha\cong\alpha$ chance to be selected, since${ N}_{-}{\gg N}_{+}$. Therefore, positive sampling will have $\alpha/(\in\times\frac{N_+}{N_-+N_+})=\frac\alpha\in\times\frac{N_-+N_+}{N_+}$ times higher probability in selecting the positive samples than uncertainty sampling. Practically, considering ${N}_{+}$ = 50,000 positive DDI or PG related abstracts, and ${N}_{-}$ = 25,000,000 negative abstracts in PubMed, a misclassification rate $\in=0.20$, and top $\alpha =20\%$ positively predicted samples are selected, positive sampling has $\frac\alpha\in\times\frac{{\text{N}}_-+{\text{N}}_+}{{\text{N}}_+}=501$ times higher chance in selecting this positive sample in AL than uncertainty sampling does.

### Active learning implementation in multiple sampling schemes in the unscreened sample pool (Fig. 1)

- *Random negative sampling and initial training and validation datasets*: According to the random negative sampling scheme, the initial training set contains 100 random negative samples from unscreened sample pool and 100 labeled positive samples from screened sample pool. Machine learning model ML1 is trained out. While the initial external validation set is made of 50 labeled negative samples, 50 positive samples and 50 random negative samples.
- *Uncertainty sampling, positive sampling, and similarity sampling*: to predict the unlabeled samples in sample pool, a random subset (100 samples) with the low confidence samples (uncertainty sampling) and high confidence positively predicted samples (positive sampling) are collected from the unscreened sample pool. In the meantime, combined with the similarity values these extracted samples are similar with the samples in corpus, the top 20 samples with high similarity are extracted and manually reviewed (similarity sampling).
- *Updating training and validation sets*: the reviewed and labeled samples from previous multiple sampling processing are divided and distributed equally into the initial training and external validation data sets. The new training set and external validation set for next round are produced.
- *Re-training*: Using the updated training set, ML_1_ is re-trained, and the multiple sampling scheme is applied again. Totally, four iterations are performed in active learning analysis.
- *Performance evaluation*: The performance of ML_1_ from all rounds of AL analysis are evaluated using the updated external validation data set.

### Active learning implementation with multiple sampling schemes in the screened sample pool (Fig. 1)

- *Datasets*: The initial training set contains 100 positive samples and 100 negative samples. Machine learning model ML_2_ is trained out. While the initial external validation set is made of 50 labeled negative samples, 50 positive samples and 50 random negative samples.
- *Uncertainty sampling and similarity sampling* : Due to AL in screened sample pool uses labeled samples as training sets, only uncertainty sampling and similarity sampling are applied to unlabeled samples in screened sample pool. After predicting the unlabeled samples in screened sample pool, a random subset (100 samples) with the low confidence samples (uncertainty sampling) are collected. Then, combined with the similarity value that extracted samples are similar with the samples in corpus, the top 20 samples with high similarity are extracted and manually reviewed (similarity sampling).
- *Updating training and validation sets*: the reviewed and labeled samples from previous multiple sampling processing are divided and distributed equally into the initial training and external validation data sets. The new training set and external validation set for next round are produced.
- *Re-training*: Using the updated training set, ML_2_ is re-trained, and the multiple sampling scheme is applied again. Totally, four iterations are performed in active learning analysis.
- *Performance evaluation*: The performance of ML_2_ from four rounds are evaluated using the updated external validation data set.

### Data preprocessing

All the abstracts are processed after downloading from PubMed. They are parsed with desired content (titles and abstracts), and are converted into GENIA format. Multiple abstract files are saved as text format in a folder. After going through Lowercase converting and StopwordsTokenizer, a Doc object for each file consisting of the text split on single space characters is transformed by basic whitespace tokenizer. This Doc is to produce to tokens that feed into models.

**Fig. 1:**
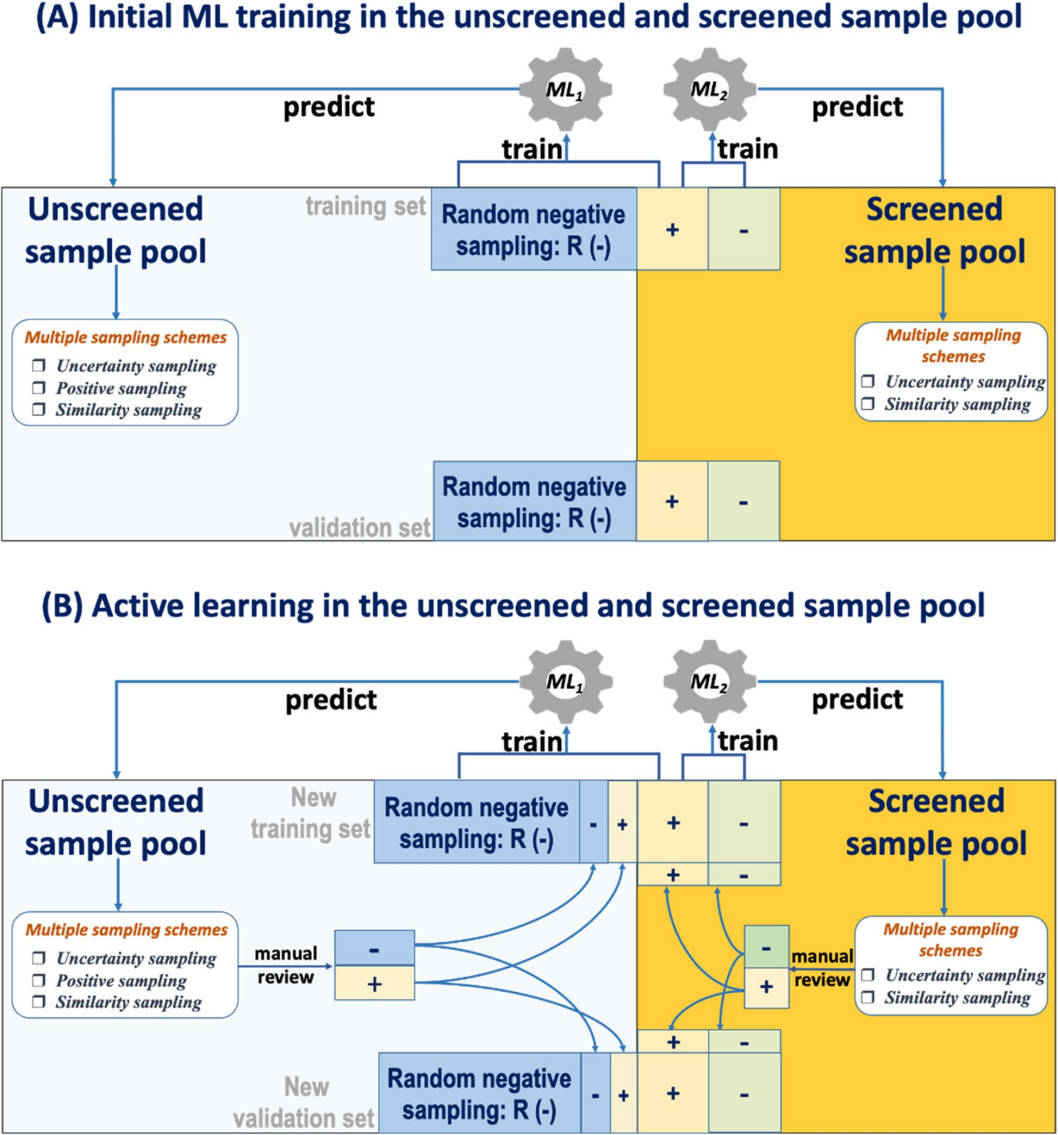
Stratified active learning with multiple sampling schemes

### Machine learning and deep learning analyses

Supporting vector machine (SVM) is used as the traditional machine learning method in AL. The appearance frequency of terms from the Doc followed Poisson distribution and was represented as a categorical term-document occurrence matrix based on the word count. The terms with low frequency SDs were considered to lack useful information and specificity. Therefore, the terms with frequency SD > 0.03 were selected as features and used to train models.

**Fig. 2:**
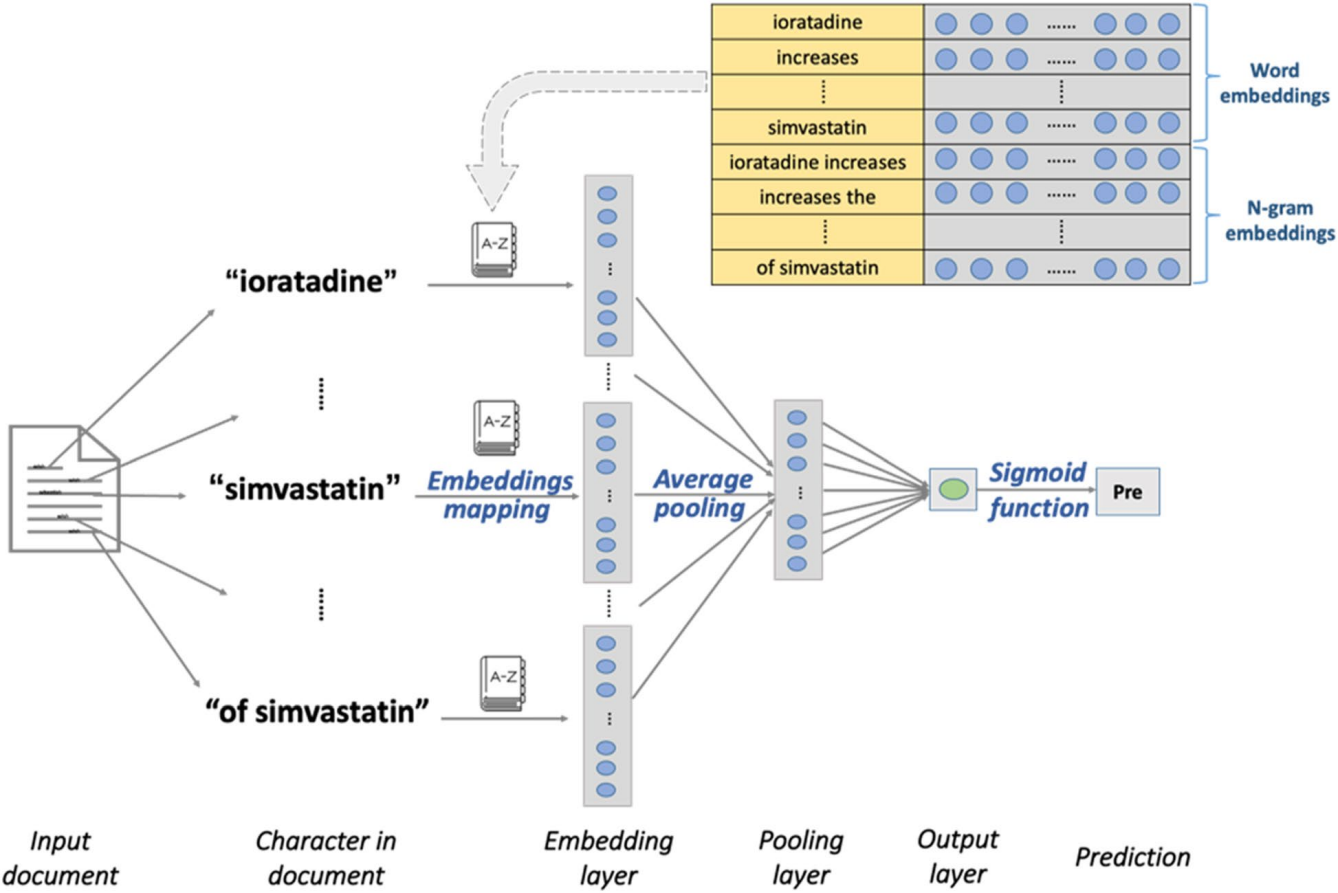
FastText scheme

FastText [39, 40] is used as a relatively simple deep learning (DL) algorithm in AL analysis. We utilize the “torch” module for text mining package in python. FastText is a multi-step approach for text classification (Fig. 2).

- *Input layer*: It is a document consisting of words, for example, “loratadine”, " increases”, " the”, " myopathy”, " risk”, " of “, “simvastatin”.
- *Embedding layer*: It maps words and the character N-grams (*N* = 2) into embedding vectors by looking up the hashed dictionary according to the global vectors (GloVe). The input words and N-grams are represented as an array that would be taken as input and extract the features.
- *Pooling layer*: A fixed-length vector by performing feature selection, the pooling layer performs element-wise averaging over all the word embeddings, followed by the output layer.
- *Softmax regression*: The sigmoid function $\varphi \left(z\right)=\frac{1}{1+{e}^{-z}}$ is used to formulate the prediction probability for an abstract: DDI positive and DDI negative.

### Performance evaluation

DDI IR AL analysis is evaluated using the following evaluation matrices: Precision (P) = TP/(TP + FP), Recall (R) = TP/(TP + FN), and the F1-score = (2*P*R)/(P + R). P is reported when R is set as 0.95. This pre-specified high recall rate serves the purpose that we will miss only a small fraction of DDI relevant paper, i.e. 0.05, in our DDI IR analysis.

## Results

### Random negative sampling plays an effective role in unscreened sample pool

**Fig. 3:**
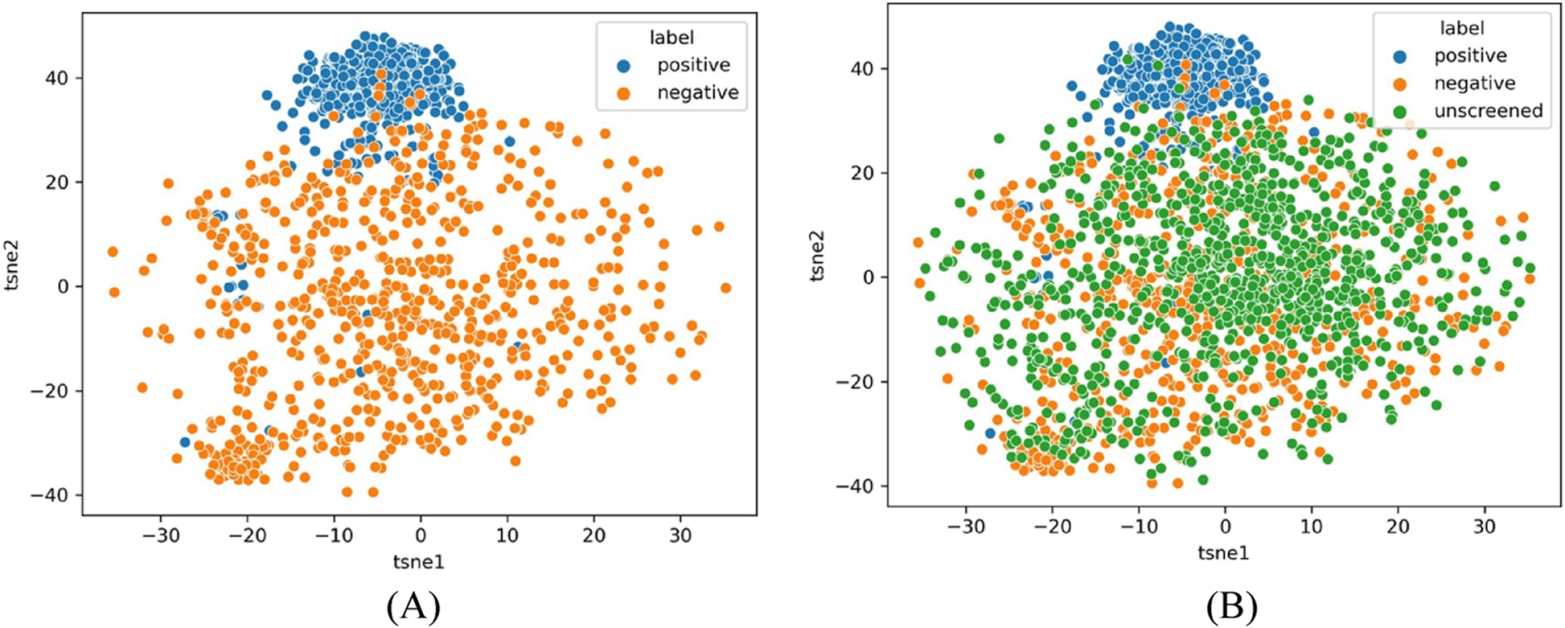
Distribution of samples from corpus and unscreened sample pool. Note: (**A**) shows the distribution of positive and negative samples in unscreened sample pool. **B** shows the positive and negative samples in the unscreened sample pool, and a random sub-sample in the unscreeed sample pool

Random negative sampling expects that DDI-related abstracts are only a very small fraction of unscreened sample pool. According to the distribution of positive and negative samples in DDI corpus, 1,000 samples are randomly selected from unscreened sample pool and on displayed. In Fig. 3, t-SNE analysis tells the distribution of most random samples from unscreened pools are the same as the negative samples’ distribution. It further illustrates that most samples in the unscreened sample pool are non-DDI related abstracts from the perspective of clustering, which makes random negative sampling more reasonable and efficient in unscreened sample pool.

#### Negatively labeled abstracts are different between screened sample pool and unscreened sample pool

**Fig. 4:**
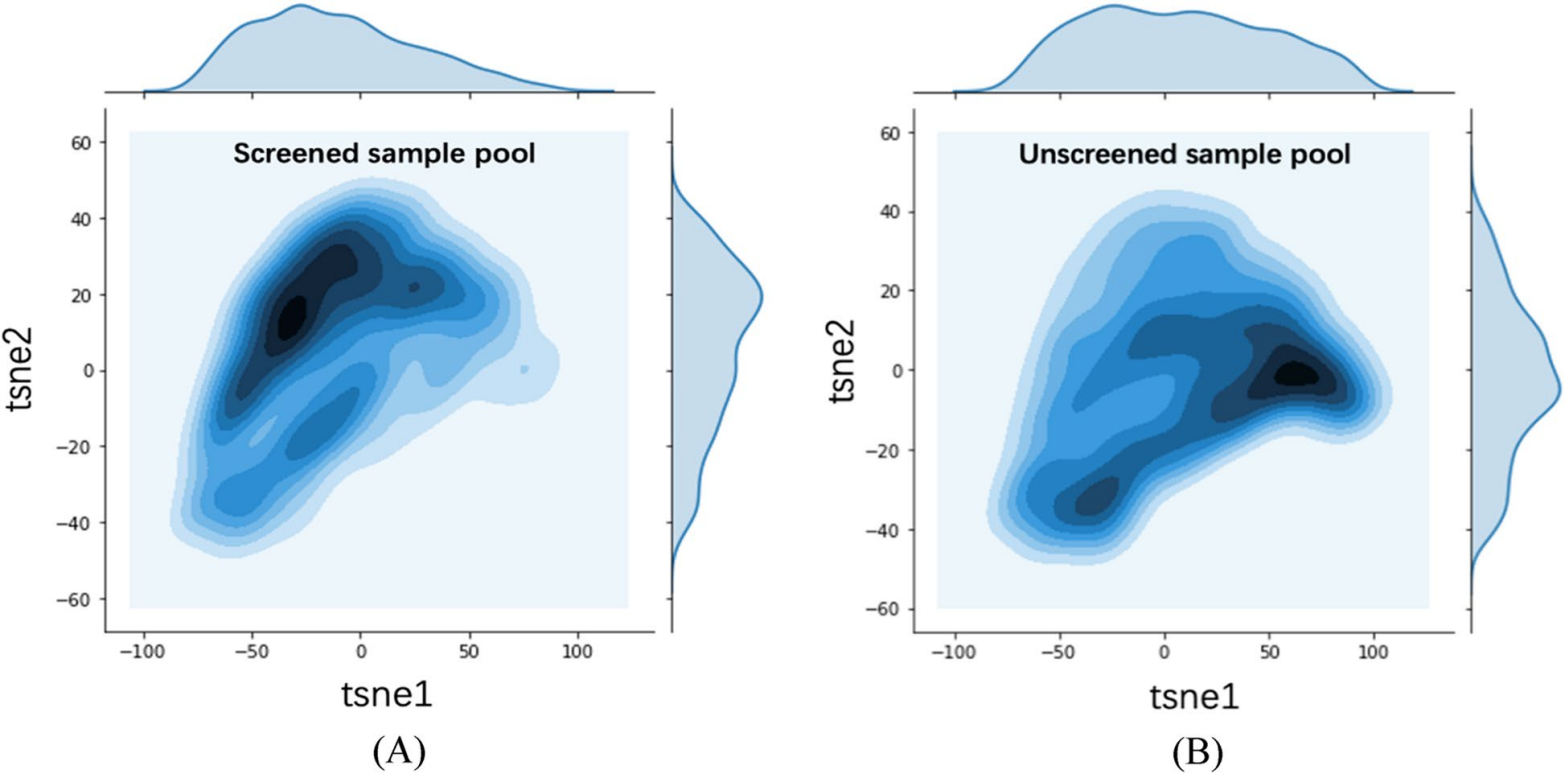
t-SNE analysis for two sample pools. Note: (**A**) distribution of negative samples in the screened sample pool; and (**B**) distribution of negative samples in the unscreened sample pool

Between screened and unscreened samples pools, we found the distribution of the negatively labeled samples are different. Using t-SNE (t-Distributed Stochastic Neighbor Embedding) visualization, it maps the high-dimensional data of abstracts to a lower dimensional space. We randomly select 300 samples in each of the two pools. Based on the data preprocessing and word embedding of the high dimensional characteristics they have, t-SNE reduced to 2 dimensions. Using the top two dimensions of t-SNE analysis, Fig. 4 shows two distributions of negatively labeled samples between the screened sample pool and the unscreened sample pool. The color in the contour plots represent the local density of the samples, and darker colored areas indicates higher density. Apparently, screened samples and unscreened samples have differently distributed negative samples. Therefore, this observation suggests that different classifiers are needed between screened sample pool and unscreened sample pool.

### Stratified AL analysis identifies more DDI relevant abstract than DDI IR analysis using only screened sample pool

PubMed comprises more than 32 million citations for biomedical literature, through the query [“drug interaction” AND (Type of Study)] and [“drug combination” AND (Type of Study)], totally 142,520 relevant literature was obtained from the year 1956 to 2021. To further verify that DDI relevant abstracts belong to very few of them, we random selected 1,000 samples from screened sample pool (pass the query) and unscreened sample pool. After manual reviewing and labeling, 25 out of 1,000 samples in screened sample pool are positive, and only 1 out of 1,220 are positive for the unscreened sample pool. It preliminary estimated that DDI relevant abstracts (positive samples) are 2.5% and 0.1% in screened and unscreened pool, respectively. Therefore, the estimated fraction of DDI relevant abstracts in two pools is about 3,563 and 26,230. Therefore, if we just use the samples in screened sample pool, we will miss potentially a large number of positive abstracts in the unscreened pool.

### Multiple sampling schemes improve the performance of AL analysis

SVM is used as the machine learning method for AL with multiple sampling schemes in screened sample pool and unscreened sample pool. In AL analysis, recall rate is all pre-specified at 0.95.

**Fig. 5:**
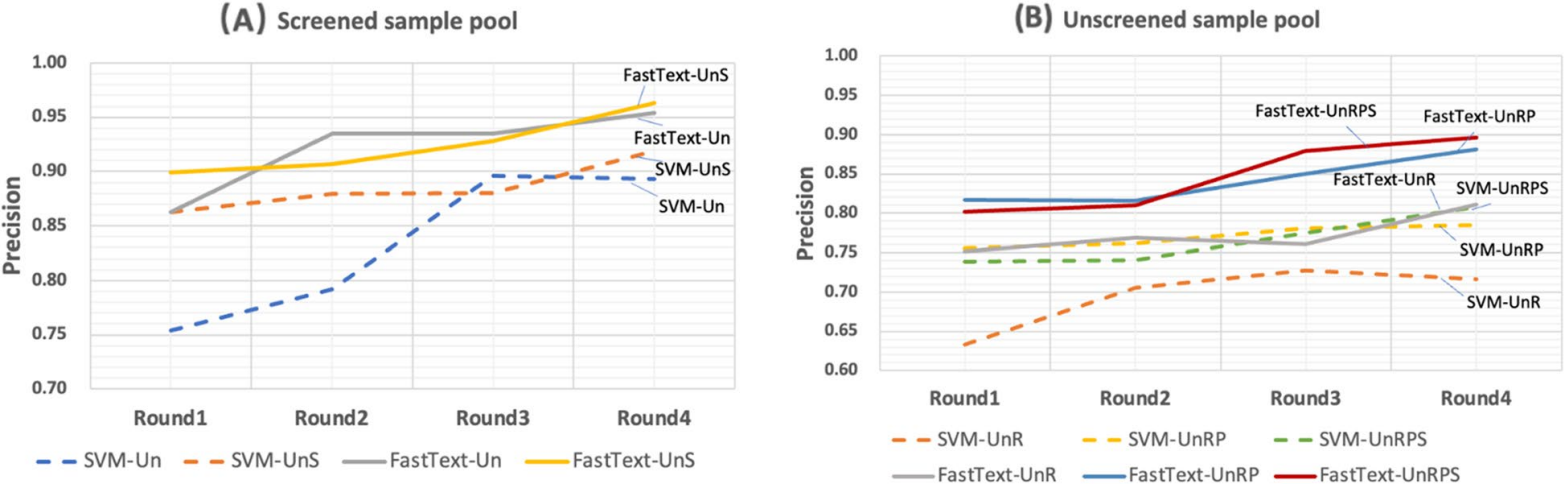
Performance of Multiple sampling in screened and unscreened sample pools. Notes:Precision in the figure presents the precision value when recall = 0.95. Un: uncertainty sampling; UnS: uncertainty sampling + similarity sampling; UnR: uncertainty sampling + random negative sampling; UnRP: uncertainty sampling + random negative sampling + positive sampling; UnRPS: uncertainty sampling + random negative sampling + positive sampling + similarity sampling
- Unscreened sample pool Fig. 5B presents the performance of three sampling schemes AL: uncertainty sampling + random negative sampling (UnR); uncertainty sampling + random negative sampling + positive sampling (UnRP); and uncertainty sampling + random negative sampling + positive sampling + similarity sampling (UnRPS). In round 1, when the recall is set at 0.95, UnRPS, UnRP, UnR have precisions of (0.75, 0.74, 0.63), respectively. Both UnRPS and UnRP out-perform UnR. UnR’s precision increases to 0.73 from round 1 to 3, but drops in round 4. However, both UnRPS and UnRP keeps stable increases in precision from round 1 to 4, and finally UnRPS has the best precision at 0.81. This analysis suggests that combined uncertainty sampling, random negative sampling, and similarity sampling leads to the best performance.

- Screened sample pool Fig. 5A compares the performance of traditional uncertainty sampling AL (Un) and AL with uncertainty sampling and similarity sampling (UnS). When recall is set as 0.95, Un keeps increasing precision from 0.75 to 0.90 from round 1 to 3 in AL analysis, until the precision performance drops in round 4. UnS, on the other hand, consistently improves the precision from round 1 to 4, from 0.86 to 0.92. This analysis demonstrates that the UnS has more steady and significant improvement of AL performance than the traditional Un method.

### Deep learning method out-performs the machine learning method in AL analysis

The performance of the embedding-based deep learning algorithm (FastText) is compared to SVM in AL analysis. Similar to the previous analysis, the recall rate is set as 0.95. The precisions are analyzed and reported in separate AL analyses from screened samples and unscreened samples.

- **Screened sample pool** Using FastText, at the beginning, i.e. round 1, FastText with UnS reaches a precision 0.90 already. It out performs FastText with Un (precision = 0.86), SVM with UnS (precision = 0.86) and SVM with Un (precision = 0.75). During AL process, FastText with either Un and UnS sampling scheme improve the precision from round 1 to 4, though Un shows a larger variation than UnS. At the end, FastText with UnS has the best precision = 0.96. These trends are shown in Fig. 5A. These data suggests that FastText, a DL method, has improved AL performance than SVM.
- **Unscreened sample pool** The performance of FastText in AL with multiple sampling schemes are compared to SVM in unscreened sample pool (Fig. 5B). At the baseline, i.e. round 1, FastText with UnRPS or UnRP have the comparable best performance, precision = 0.80 and 0.81, respectively. Their precision steadily improve from round 1 to 4, and reach to 0.90 and 0.88, respectively. These numbers are noticeably higher than those from SVM method with multiple sampling schemes.

## Discussion

This study performed a comprehensive investigation on how various sampling schemes and machine learning algorithms improve AL for DDI IR analysis from literature. This is also the first time that AL is studied for its performance in DDI IR analysis. DDI IR analysis from PubMed abstracts faces the challenges of relatively small positive DDI samples and overwhelmingly large negative samples. New sampling schemes, including random negative sampling and positive sampling, are purposely designed to address these challenges. They reduce annotation labor and improve the efficiency of AL analysis. The theoretical consistency of random negative sampling and positive sampling is also shown in the paper.

Practically, PubMed abstracts are divided into two pools. Screened pool contains all abstracts that pass the DDI keywords query in PubMed, while unscreened pool includes all the other abstracts. Our preliminary analysis reveals that the unscreened pool contains seven times more DDI related abstracts, 26,230, than the screened pool, 3563. This shows that we cannot only rely on PubMed query in retrieve DDI related abstracts.

At a prespecified recall rate of 0.95, DDI IR analysis performance is evaluated and compared in precision. In screened pool IR analysis using supporting vector machine (SVM), similarity sampling plus uncertainty sampling improves the precision of AL over uncertainty sampling, from 0.89 to 0.92 respectively. In the unscreened pool IR analysis, the integrated random negative sampling, positive sampling, and similarity sampling improve the IR analysis performance over uncertainty sampling along, from 0.72 to 0.81 respectively. When we change the SVM to a deep learning method, all sampling schemes consistently benefit DDI AL analysis in both screened pool and unscreened pool. Deep learning also has significant improvement of precision over SVM, 0.96 vs. 0.91 in screened pool, and 0.90 vs. 0.81 in the unscreened pool, respectively. Please note that the recall is all set 0.95 for all occasions in our IR analysis. The 0.96 and 0.90 precision performance are extraordinary.

Random negative sampling and positive sampling are effective methods in improving AL analysis when a sample pool is dominated with negative samples. In our DDI IR analysis, they effectively reduce the annotation workload, and improve the IR analysis performance. We believe these two sampling schemes are equally effective to other NLP applications where the positive and negative samples are imbalanced.

Similarity sampling can be a two-edged sword. If the initial samples are biased samples from the sample pool, similarity sampling will lead to biased samples, hence mis-trained machine learning models. On the other hand, uncertainty sampling itself can introduce a large variation in each individual sampling step, such that new samples can be highly different from original samples, and the convergence of active learning algorithm becomes questionable. This is where similarity sampling can effectively reduce the variability in active learning. We compared the active learning performance with or without similarity sampling in both screened sample pool and unscreened sample pool, and under two machine model, SVM and FastText (Supplementary Figures S1-S2). We repeated the activity learning five different times independently. We can see similarity sampling significantly reduces the variation, and improves the convergency.

The least confidence sampling was not the only uncertainty sampling scheme in active learning, we also investigated two other uncertainty sampling schemes, named margin sampling [41] and entropy [42]. They have provided comparable performance (see Supplementary Figures S3-S4).

## Conclusion

This paper developed multiple sampling schemes and deep learning algorithms, and implemented them in the active learning (AL). This is the first time that AL is developed to preform drug-drug interaction information retrieval (DDI IR) analysis. The superior performance of deep learning to the conventional machine learning approaches is a major conclusion in AL DDI IR analysis. We further demonstrate that both positive sampling and random negative sampling schemes are highly effective sampling scheme in AL analysis, when positive samples are extremely small and negative samples are overwhelmingly large.

## Supplementary Information

**Additional file 1: Figure S1.** Performance of uncertainty AQsampling + similarity sampling in screened sample pools. **Figure S2.** Performance of uncertainty sampling + similarity sampling in unscreened sample pools. **Figure S3.** Performance of margin-based sampling. **Figure S4.** Performance of Entropy-based sampling.

## Data Availability

The datasets generated and analyzed during the current study are available in GitHub at https://github.com/zha204/Deep-AL-for-DDI-IR.

## Abbreviations

DDI: drug-drug interaction
AL: active learning
ADEs: adverse drug events
ML: machine learning
DL: deep learning
IR: information retrieval
IE: information extraction
PK: pharmacokinetics
PE: pharmaco-epidemiology
SVM: supporting vector machine.
