# Supplementary material for: Multiple sampling schemes and deep learning improve active learning performance in drug-drug interaction information retrieval analysis from the literature
Source: J Biomed Semantics. 2023 May 30;14:5. doi: 10.1186/s13326-023-00287-7 (PMC10228061; doi:10.1186/s13326-023-00287-7)
Supplement: Supplementary file 1 — Additional file 1: Figure S1. Performance of uncertainty AQsampling + similarity sampling in screened sample pools. Figure S2. Performance of uncertainty sampling + similarity sampling in unscreened sample pools. Figure S3. Performance of margin-based sampling. Figure S4. Performance of Entropy-based sampling. [file 13326_2023_287_MOESM1_ESM.docx]

Supplementary Figures:

| **Figure S1: Performance of uncertainty sampling + similarity sampling in screened sample pools** |
| --- |
| 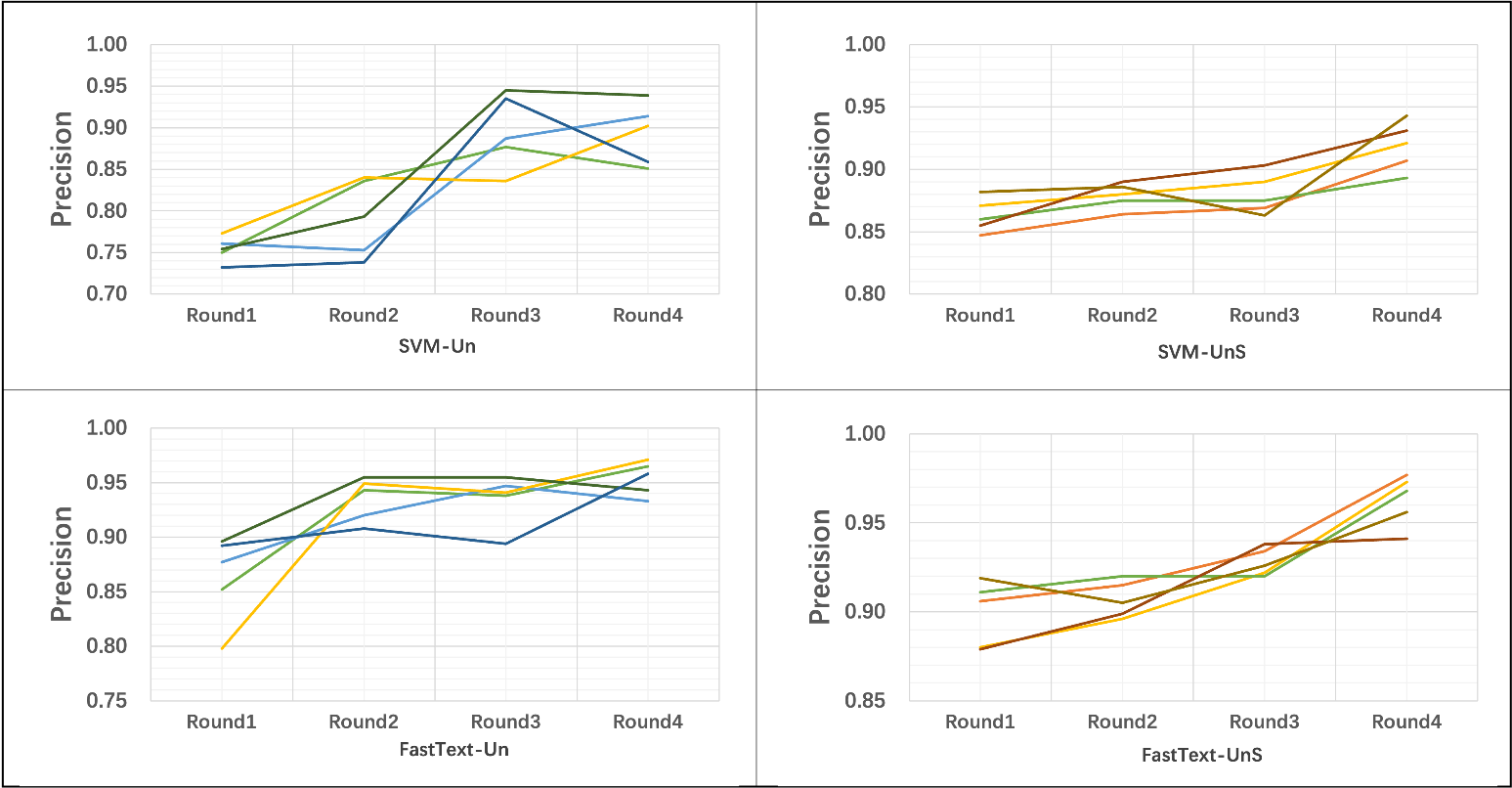 |
| **Figure S2: Performance of uncertainty sampling + similarity sampling in unscreened sample pools** |
| 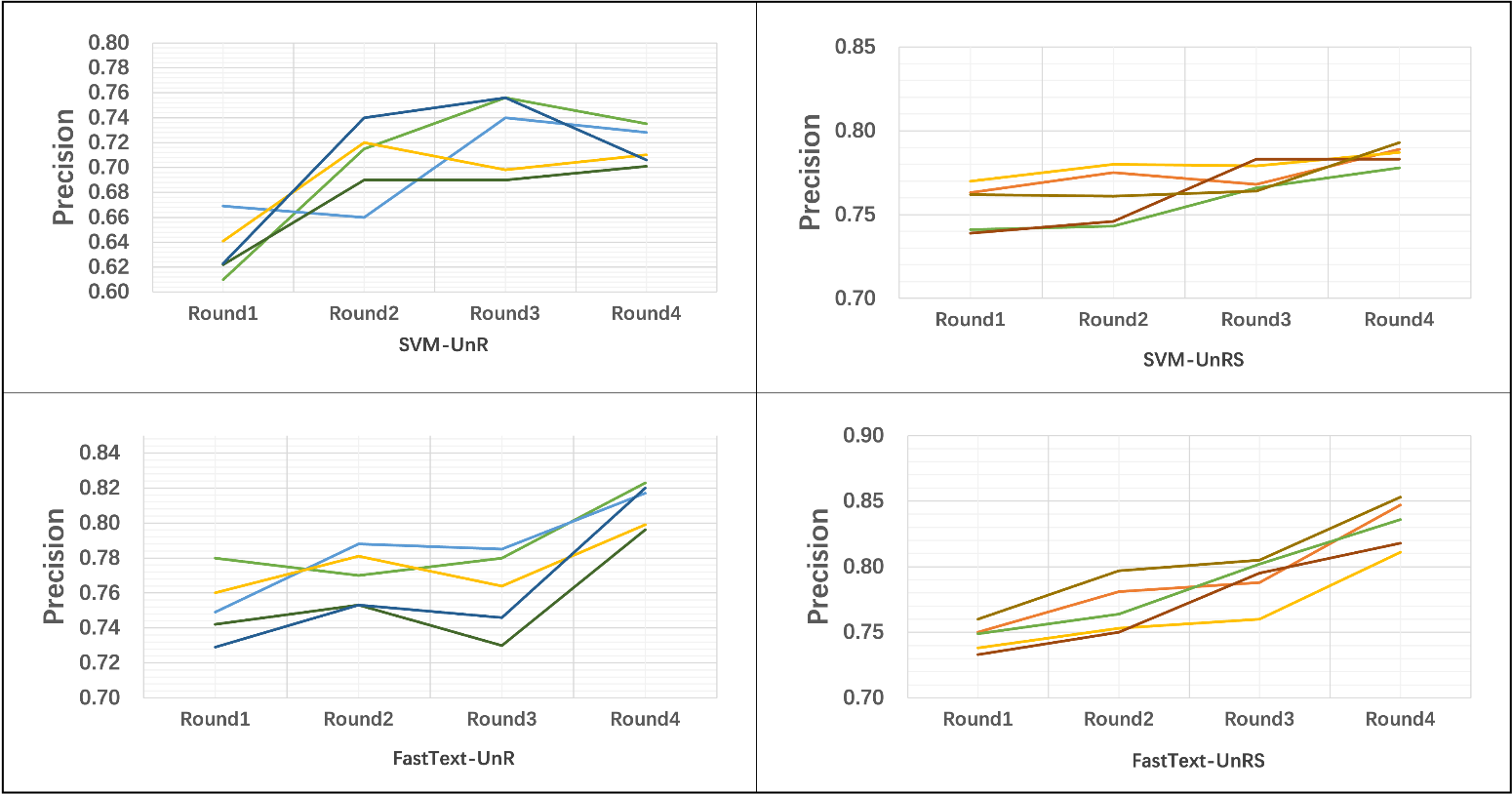 |
| ***Notes:*** *Precision in the figure presents the precision value when recall = 0.95. The different lines in each plot represent the repetitions.*  ***Un****: uncertainty sampling;*  ***UnS****: uncertainty sampling + similarity sampling;*  ***UnR****: uncertainty sampling + random negative sampling + positive sampling*  ***UnRS****: uncertainty sampling + random negative sampling + positive sampling + similarity sampling.* |

| **Figure S3: Performance of margin-based sampling** |
| --- |
| **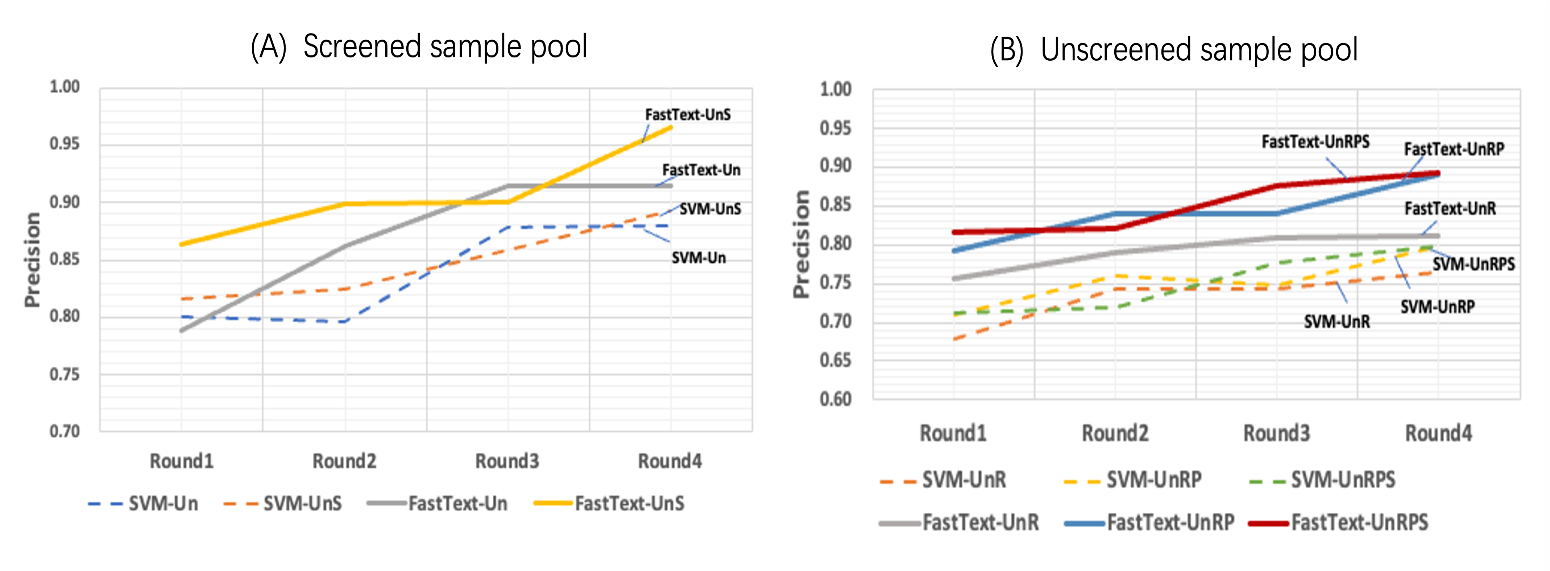** |
| **Figure S4: Performance of Entropy-based sampling** |
| 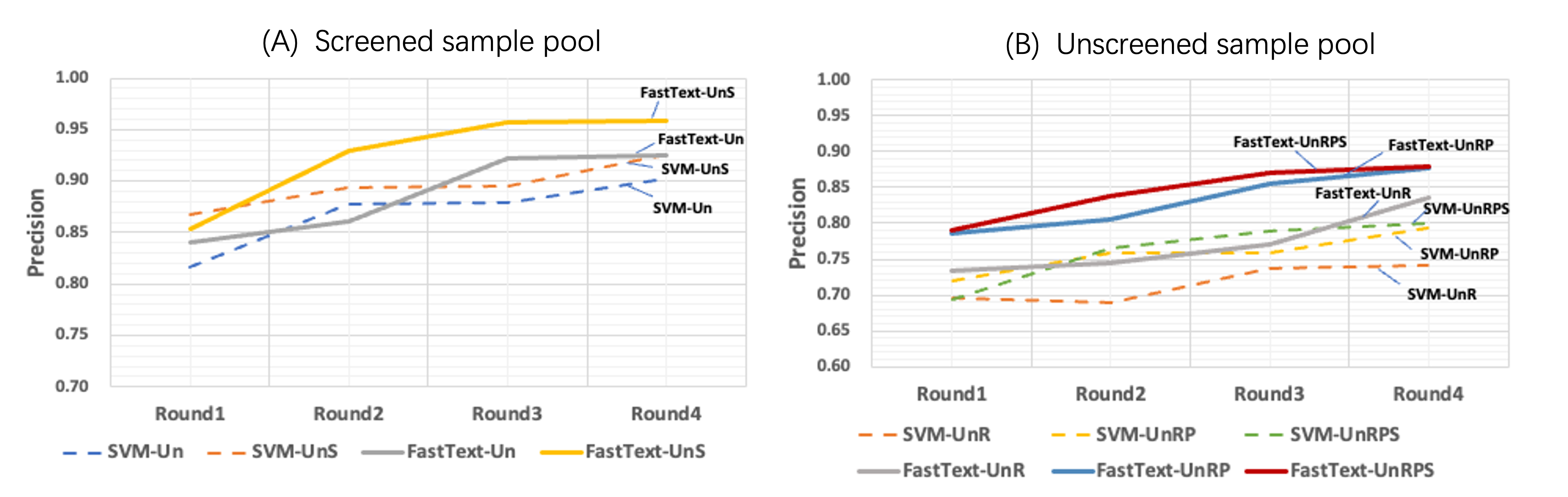 |
| ***Notes:*** *Precision in the figure presents the precision value when recall = 0.95.*  *Un: uncertainty sampling;*  *UnS: uncertainty sampling + similarity sampling;*  *UnR: uncertainty sampling + random negative sampling;*  *UnRP: uncertainty sampling + random negative sampling + positive sampling;*  *UnRPS: uncertainty sampling + random negative sampling + positive sampling + similarity sampling.* |
